# Crystal structure of poly[(4-amino­pyridine-κ*N*)(*N*,*N*-di­methyl­formamide-κ*O*)(μ_3_-pyridine-3,5-di­carboxyl­ato-κ^3^ *N*:*O* ^3^:*O* ^5^)copper(II)]

**DOI:** 10.1107/S205698901600342X

**Published:** 2016-03-04

**Authors:** Cheng-Chen Shen, Xiu-Ni Hua, Lei Han

**Affiliations:** a Institute of Inorganic Materials, School of Materials Science and Chemical Engineering, Ningbo University, Ningbo, Zhejiang 315211, People’s Republic of China

**Keywords:** crystal structure, metal–organic framework, chiral network, (10,3)-*a* topology

## Abstract

The title compound, [Cu(C_7_H_3_NO_4_)(C_5_H_6_N_2_)(C_3_H_7_NO]_*n*_, is an amino-function­alized chiral metal–organic framework with (10,3)-*a* topology. It has been constructed *via* the assembly of the achiral triconnected pyridine-3,5-di­carboxyl­ate (3,5-PDC) building block and a triconnected Cu^II^ atom. Each Cu^II^ ion is coordinated by two O atoms and one N atom, respectively, of three crystallographically independent 3,5-PDC ligands. The square-pyramidal (CuN_2_O_3_) coordination geometry of the Cu^II^ ion is completed by an N atom of a terminal 4-amino­pyridine (4-APY) ligand and the O atom of a terminal *N*,*N*-di­methyl­formamide (DMF) ligand to give a triconnected ‘T’-shaped secondary building unit, which becomes trigonal in the resulting (10,3)-*a* topology. In the three-dimensional structure, weak N—H⋯O hydrogen bonds are observed in which the donor N—H groups are provided by the 4-APY ligands and the acceptor O atoms are provided by the non-coordinating carboxylate O atoms of the 3,5-PDC ligands.

## Abstract 2

An amino-functionalized chiral metal–organic framework with (10,3)-*a* topology has been constructed *via* the assembly of the achiral triconnected building block pyridine-3,5-di­carboxyl­ate and a triconnected Cu^II^ centre.

## Chemical context   

Research on metal–organic frameworks (MOFs) has attracted much attention in recent years not only for their great potential applications, such as in gas storage, separation, fluorescence and magnetism, but also for their intriguing topologies and structural diversity (Allendorf *et al.*, 2009 ▸). Of special inter­est is the rational design and synthesis of chiral networks, which offer great potential in non-linear optics, asymmetric catalysis, and chiral separation (Evans & Lin, 2002 ▸; Zhang & Xiong, 2012 ▸). Therefore, a logical target for synthesis would be a default structure that possesses chirality. The (10,3)-*a* network meets these requirements since it is mutually chiral and regarded as the default three-dimensional structure for the assembly of triconnected building blocks (Eubank *et al.*, 2005 ▸; Han *et al.*, 2013*a* ▸).

On the other hand, amino-functionalized porous metal-organic frameworks have also attracted much attention. Recent research on amino-functionalized MOFs revealed that they have high CO_2_ adsorption capacity at lower pressure due to the potential inter­action between amino groups and CO_2_ (Couck *et al.*, 2009 ▸). Amino-functionalized MOFs can also act as reaction active sites for the post-synthesis modification of metal-organic frameworks (Shultz *et al.*, 2011 ▸).

As a continuation of our group research on the assembly of amino-functionalized chiral metal–organic frameworks (Han *et al.*, 2011 ▸, 2013*b* ▸; Pan *et al.*, 2014 ▸), we herein report the preparation and crystal structure of Cu(3,5-PDC)(4-APY)(DMF), (3,5-PDC = pyridine-3,5-di­carboxyl­ate, 4-APY = 4-amino­pyridine, DMF = *N*,*N*-di­methyl­formamide), which was constructed *via* the assembly of the achiral triconnected building block pyridine-3,5-di­carboxyl­ate (3,5- PDC) and a triconnected Cu^II^ atom, CuN(CO_2_)_2_, synthesized *in situ*. The title compound is an inter­esting example of an amino-functionalized chiral metal-organic framework with (10,3)-*a* topology assembled from achiral ligands. This amino-functionalized chiral framework can be used for depositing small gold nanoparticles using a solution-based adsorption/reduction preparation method, and offer myriad opportunities for chiral catalysis.
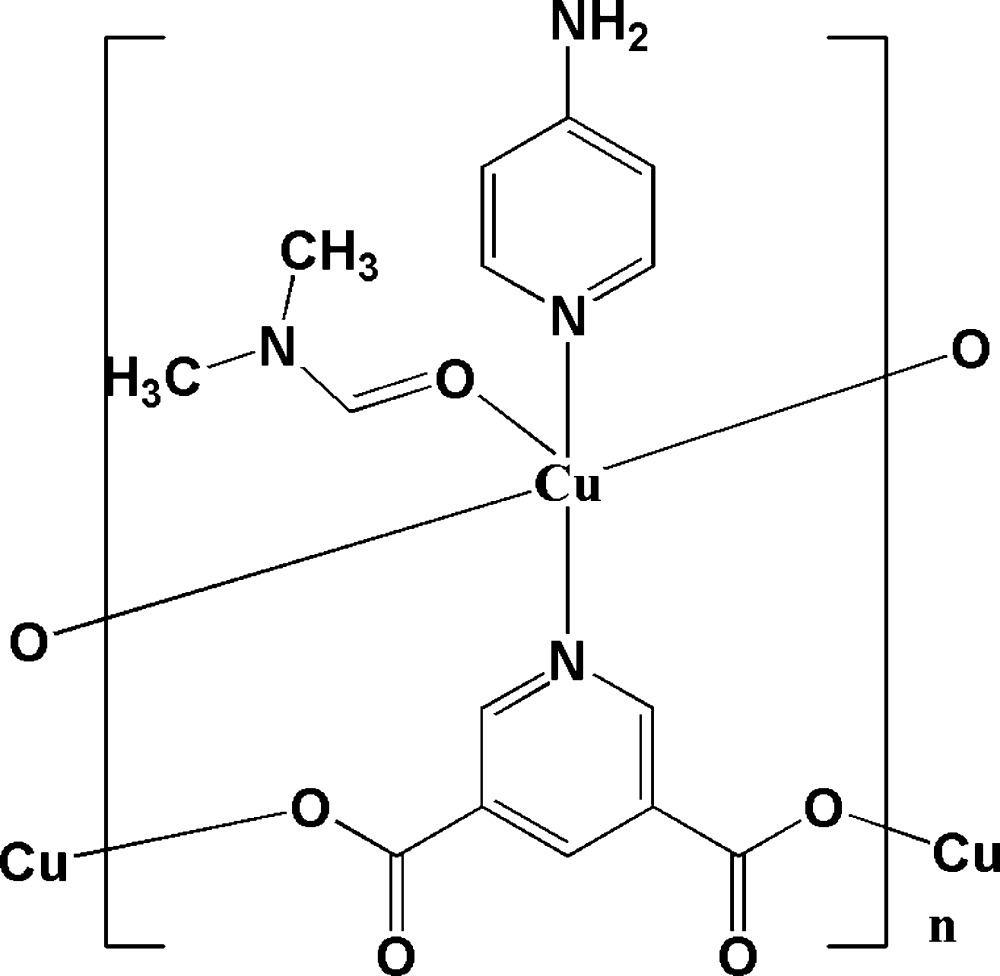


## Structural commentary   

**Figure 1:**
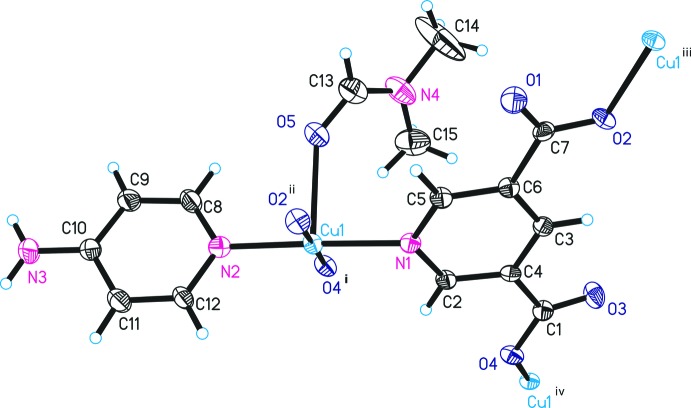
The asymmetric unit of title compound, showing the atom-numbering scheme. Displacement ellipsoids are drawn at the 30% probability level. [Symmetry codes: (i) *x* − 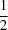, −*y* + 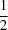, −*z* + 1; (ii) −*x*, *y* − 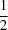, −*z* + 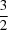; (iii) −*x*, *y* + 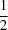, −*z* + 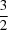; (iv) *x* + 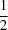, −*y* + 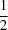, −*z* + 1.]

The asymmetric unit of the title compound, Cu(3,5-PDC)(4-APY)(DMF), contains one Cu^II^ ion, one 3,5-PDC anion, one 4-apy mol­ecule and one DMF mol­ecule. As shown in Fig. 1 ▸, each Cu^II^ ion adopts a square-pyramidal (CuN_2_O_3_) coordin­ation geometry. In the equatorial plane, the Cu^II^ ion is coord­inated by two oxygen atoms and one nitro­gen atom, respectively, of three crystallographically independent 3,5-PDC ligands, and one nitro­gen atom of a terminal 4-APY ligand. The oxygen atom of a terminal DMF mol­ecule is bonded to the Cu^II^ ion in the axial position to complete the square-pyramidal coordination geometry. The bond lengths and bond angles around the Cu^II^ ion are in good agreement with similar structures (Eubank *et al.*, 2005 ▸; Lu *et al.*, 2006 ▸). The axial Cu—O_DMF_ bond length [2.396 (4) Å] is longer than the equatorial Cu—O_carboxyl­ate_ and Cu—N_4-APY_ bonds due to the Jahn–Teller effect of the Cu^2+^ atom.

**Figure 2:**
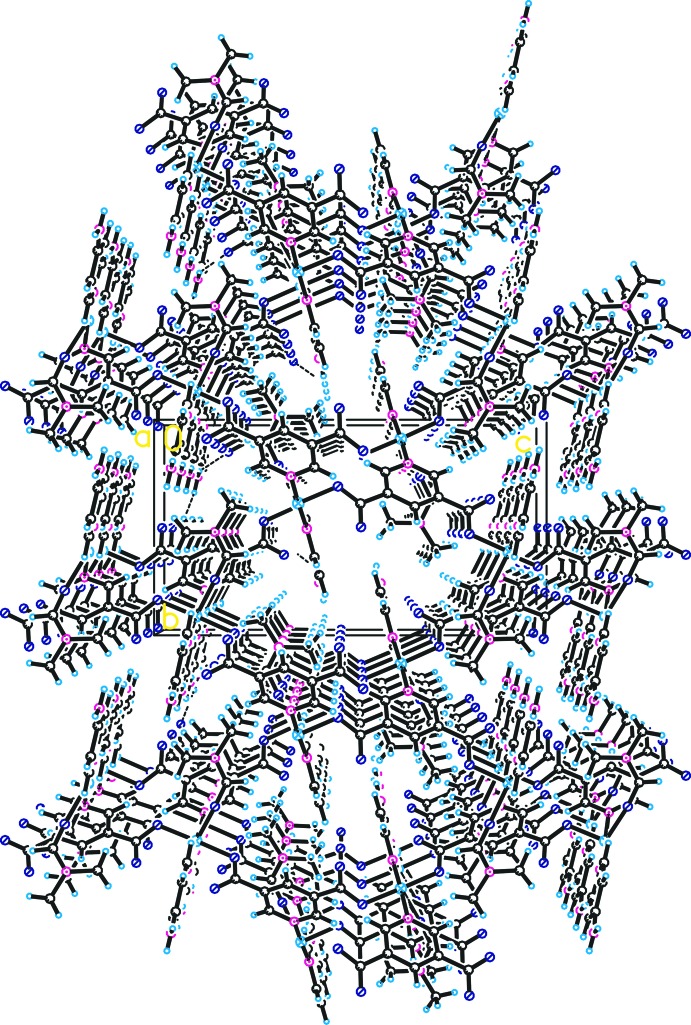
Crystal packing of the title compound viewed along the *a* axis, showing hydrogen bonds as dashed lines.

**Figure 3:**
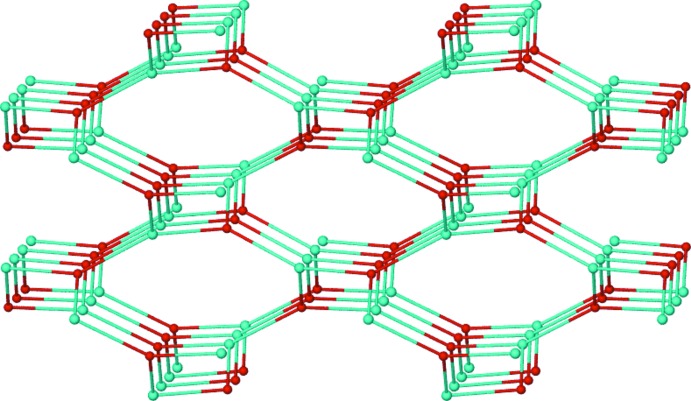
A representation of the (10,3)-*a* topology.

The three-dimensional structure of the title compound viewed along the *a* axis is shown in Fig. 2 ▸. To analyse the topology, the square-pyramidal coordination geometry of copper can act as a ‘T’-shaped triconnected secondary building unit (Fig. 2 ▸), which becomes trigonal in the resulting topology. At the same time, 3,5-PDC acts as another triconnected node since it possesses two deprotonated carb­oxy­lic acid coordin­ating sites, and a third, neutral aromatic nitro­gen coordinating site. As a result, the desired triconnected (10,3)-*a* network is obtained, as shown in Fig. 3 ▸. The terminally coordinated 4-APY and DMF ligands are oriented to the inter­ior of the channels and thus prevent self-inter­penetration. The (10,3)-*a* topology leads to an enanti­opure network of the title compound (Eubank *et al.*, 2005 ▸; Han *et al.*, 2013*a* ▸), despite being formed solely from achiral mol­ecular units.

## Supra­molecular features   

By introducing 4-amino­pyridine as co-ligand, the amino-functionalized chiral metal-organic framework was successfully designed and synthesized. Additionally, the –NH_2_ group of the 4-APY ligand can act as the donor N—H groups to form hydrogen bonds (Han *et al.*, 2011 ▸). In the three-dimensional structure of the title compound, weak N—H⋯O hydrogen bonds are observed (Table 1 ▸) in which the acceptors are provided by the non-coordinating oxygen atoms of the carboxyl­ate groups of the 3,5-PDC ligands.

## Synthesis and crystallization   

The title compound was prepared by a solvothermal method. A mixture of pyridine-3,5-di­carb­oxy­lic acid (0.0339 g, 0.2 mmol), 4-amino­pyridine (0.0098 g, 0.10 mmol) and Cu(NO_3_)_2_·3H_2_O (0.0484 g, 0.20 mmol) in 6 ml DMF solution was stirred at room temperature for 30 minutes, and subsequently sealed in a 25 ml Teflon-lined stainless steel reactor. The reactor was heated at 363 K for 3 d. A crop of blue, block-shaped single crystals of the title compound was obtained after cooling the solution to room temperature. The yield was approximately 70% based on Cu salt.

## Refinement details   

Crystal data, data collection and structure refinement details are summarized in Table 2 ▸. All H atoms were placed in geometrically idealized positions and refined in a riding-model approximation on their parent atoms, with *U*_iso_(H) = 1.2*U*_eq_(C) (aromatic) and 1.5*U*_eq_(C) (meth­yl) with C—H = 0.93 Å (aromatic) and 0.96 Å (meth­yl), and *U*_iso_(H) = 1.2*U*_eq_(N) with N—H = 0.86 Å.

## Supplementary Material

Crystal structure: contains datablock(s) I. DOI: 10.1107/S205698901600342X/lh5806sup1.cif

Structure factors: contains datablock(s) I. DOI: 10.1107/S205698901600342X/lh5806Isup2.hkl

CCDC reference: 1456364

Additional supporting information:  crystallographic information; 3D view; checkCIF report

## Figures and Tables

**Table 1:** Hydrogen-bond geometry (Å, °)

*D*—H⋯*A*	*D*—H	H⋯*A*	*D*⋯*A*	*D*—H⋯*A*
N3—H3*A*⋯O3^i^	0.86	2.28	3.101 (7)	159
N3—H3*B*⋯O1^ii^	0.86	2.23	2.933 (7)	139

Symmetry codes: (i) 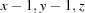; (ii) 
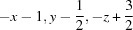
.

**Table 2:** Experimental details

Crystal data	
Chemical formula	[Cu(C_7_H_3_NO_4_)(C_5_H_6_N_2_)(C_3_H_7_NO)]
*M*_r_	395.86
Crystal system, space group	Orthorhombic, *P*2_1_2_1_2_1_
Temperature (K)	293
*a*, *b*, *c* (Å)	8.3365 (17), 10.453 (2), 19.030 (4)
*V* (Å^3^)	1658.2 (6)
*Z*	4
Radiation type	Mo *K*α
μ (mm^−1^)	1.35
Crystal size (mm)	0.24 × 0.20 × 0.19

Data collection	
Diffractometer	Bruker APEXII DUO CCD
Absorption correction	Analytical [based on measured indexed crystal faces using *SHELXL2014* (Sheldrick, 2015*b* ▸)]
*T*_min_, *T*_max_	0.716, 0.773
No. of measured, independent and observed [*I* > 2σ(*I*)] reflections	16376, 3799, 2926
*R*_int_	0.051
(sin θ/λ)_max_ (Å^−1^)	0.649

Refinement	
*R*[*F* ^2^ > 2σ(*F* ^2^)], *wR*(*F* ^2^), *S*	0.039, 0.132, 1.15
No. of reflections	3799
No. of parameters	226
H-atom treatment	H-atom parameters constrained
Δρ_max_, Δρ_min_ (e Å^−3^)	0.79, −1.17
Absolute structure	Flack (1983 ▸), 1619 Friedel pairs
Absolute structure parameter	0.00 (2)

Computer programs: *APEX2* and *SAINT* (Bruker, 2014 ▸), *SHELXT* (Sheldrick, 2015*a* ▸), *SHELXL2014* (Sheldrick, 2015*b* ▸), *XP* in *SHELXTL-Plus* (Sheldrick, 2008 ▸) and *publCIF* (Westrip, 2010 ▸).

## supplementary crystallographic information

### Crystal data

**Table:**

[Cu(C_7_H_3_NO_4_)(C_5_H_6_N_2_)(C_3_H_7_NO)]	*D*_x_ = 1.586 Mg m^−^^3^
*M**_r_* = 395.86	Mo *K*α radiation, λ = 0.71073 Å
Orthorhombic, *P*2_1_2_1_2_1_	Cell parameters from 2974 reflections
*a* = 8.3365 (17) Å	θ = 3.1–27.5°
*b* = 10.453 (2) Å	µ = 1.35 mm^−^^1^
*c* = 19.030 (4) Å	*T* = 293 K
*V* = 1658.2 (6) Å^3^	Block, blue
*Z* = 4	0.24 × 0.20 × 0.19 mm
*F*(000) = 812	

### Data collection

**Table:**

Bruker APEXII DUO CCD diffractometer	3799 independent reflections
Radiation source: fine-focus sealed tube	2926 reflections with *I* > 2σ(*I*)
Graphite monochromator	*R*_int_ = 0.051
ω–scans	θ_max_ = 27.5°, θ_min_ = 3.1°
Absorption correction: analytical [based on measured indexed crystal faces using *SHELXL2014* (Sheldrick, 2015*b*)]	*h* = −10→10
*T*_min_ = 0.716, *T*_max_ = 0.773	*k* = −13→13
16376 measured reflections	*l* = −24→22

### Refinement

**Table:**

Refinement on *F*^2^	Secondary atom site location: difference Fourier map
Least-squares matrix: full	Hydrogen site location: inferred from neighbouring sites
*R*[*F*^2^ > 2σ(*F*^2^)] = 0.039	H-atom parameters constrained
*wR*(*F*^2^) = 0.132	*w* = 1/[σ^2^(*F*_o_^2^) + (0.0573*P*)^2^ + 1.9518*P*] where *P* = (*F*_o_^2^ + 2*F*_c_^2^)/3
*S* = 1.15	(Δ/σ)_max_ = 0.013
3799 reflections	Δρ_max_ = 0.79 e Å^−^^3^
226 parameters	Δρ_min_ = −1.16 e Å^−^^3^
0 restraints	Absolute structure: Flack (1983), 1619 Friedel pairs
Primary atom site location: structure-invariant direct methods	Absolute structure parameter: 0.00 (2)

### Special details

**Table:**

Geometry. All e.s.d.'s (except the e.s.d. in the dihedral angle between two l.s. planes) are estimated using the full covariance matrix. The cell e.s.d.'s are taken into account individually in the estimation of e.s.d.'s in distances, angles and torsion angles; correlations between e.s.d.'s in cell parameters are only used when they are defined by crystal symmetry. An approximate (isotropic) treatment of cell e.s.d.'s is used for estimating e.s.d.'s involving l.s. planes.
Refinement. Refinement of *F*^2^ against ALL reflections. The weighted *R*-factor *wR* and goodness of fit *S* are based on *F*^2^, conventional *R*-factors *R* are based on *F*, with *F* set to zero for negative *F*^2^. The threshold expression of *F*^2^ > σ(*F*^2^) is used only for calculating *R*-factors(gt) *etc*. and is not relevant to the choice of reflections for refinement. *R*-factors based on *F*^2^ are statistically about twice as large as those based on *F*, and *R*- factors based on ALL data will be even larger.

### Fractional atomic coordinates and isotropic or equivalent isotropic displacement parameters (Å^2^)

**Table:**

	*x*	*y*	*z*	*U*_iso_*/*U*_eq_	
Cu1	−0.23946 (7)	0.10503 (5)	0.62338 (2)	0.02797 (16)	
O1	0.0648 (5)	0.3766 (4)	0.83488 (17)	0.0497 (10)	
O2	0.2102 (4)	0.5278 (3)	0.78331 (16)	0.0348 (8)	
O3	0.3228 (5)	0.4977 (4)	0.5253 (2)	0.0498 (11)	
O4	0.2342 (5)	0.3222 (3)	0.47057 (14)	0.0337 (7)	
O5	−0.4048 (6)	0.2700 (4)	0.6723 (3)	0.0605 (12)	
N1	−0.0413 (5)	0.2178 (4)	0.63928 (18)	0.0284 (8)	
N2	−0.4300 (5)	−0.0057 (4)	0.6043 (2)	0.0347 (10)	
N3	−0.8209 (6)	−0.2450 (6)	0.5764 (3)	0.0705 (18)	
H3A	−0.8050	−0.3239	0.5657	0.085*	
H3B	−0.9170	−0.2170	0.5822	0.085*	
N4	−0.3401 (8)	0.4804 (5)	0.6705 (3)	0.0625 (17)	
C1	0.2489 (6)	0.3962 (4)	0.5239 (2)	0.0315 (9)	
C2	0.0476 (6)	0.2565 (4)	0.5840 (2)	0.0291 (10)	
H2A	0.0301	0.2180	0.5406	0.035*	
C3	0.1883 (6)	0.4086 (5)	0.6536 (2)	0.0297 (10)	
H3C	0.2624	0.4745	0.6582	0.036*	
C4	0.1634 (6)	0.3505 (4)	0.5889 (2)	0.0267 (9)	
C5	−0.0125 (6)	0.2721 (5)	0.7013 (2)	0.0310 (10)	
H5A	−0.0716	0.2450	0.7400	0.037*	
C6	0.1011 (6)	0.3670 (4)	0.7112 (2)	0.0278 (10)	
C7	0.1250 (6)	0.4263 (5)	0.7828 (2)	0.0316 (11)	
C8	−0.5796 (7)	0.0376 (5)	0.6086 (3)	0.0448 (13)	
H8A	−0.5945	0.1240	0.6183	0.054*	
C9	−0.7126 (7)	−0.0366 (5)	0.5999 (3)	0.0502 (15)	
H9A	−0.8141	−0.0008	0.6045	0.060*	
C10	−0.6962 (7)	−0.1657 (6)	0.5839 (3)	0.0458 (14)	
C11	−0.5405 (7)	−0.2092 (5)	0.5747 (3)	0.0521 (15)	
H11A	−0.5225	−0.2933	0.5608	0.062*	
C12	−0.4130 (7)	−0.1294 (5)	0.5860 (3)	0.0454 (13)	
H12A	−0.3100	−0.1621	0.5808	0.055*	
C13	−0.3972 (9)	0.3755 (6)	0.7001 (4)	0.0610 (17)	
H13A	−0.4348	0.3824	0.7460	0.073*	
C14	−0.330 (2)	0.5962 (7)	0.7100 (5)	0.164 (7)	
H14A	−0.3715	0.5821	0.7564	0.246*	
H14B	−0.3920	0.6616	0.6872	0.246*	
H14C	−0.2201	0.6229	0.7130	0.246*	
C15	−0.2832 (11)	0.4792 (7)	0.5991 (4)	0.076 (2)	
H15A	−0.2989	0.3957	0.5793	0.114*	
H15B	−0.1711	0.5001	0.5983	0.114*	
H15C	−0.3418	0.5411	0.5720	0.114*	

### Atomic displacement parameters (Å^2^)

**Table:**

	*U*^11^	*U*^22^	*U*^33^	*U*^12^	*U*^13^	*U*^23^
Cu1	0.0355 (3)	0.0291 (3)	0.0193 (2)	−0.0032 (3)	−0.0035 (2)	0.0024 (2)
O1	0.054 (2)	0.074 (3)	0.0212 (16)	−0.017 (2)	0.0063 (16)	−0.0070 (18)
O2	0.044 (2)	0.0349 (16)	0.0254 (15)	−0.0002 (16)	−0.0046 (15)	−0.0092 (14)
O3	0.064 (3)	0.049 (2)	0.0366 (19)	−0.0273 (19)	0.0124 (19)	−0.0012 (17)
O4	0.051 (2)	0.0313 (15)	0.0188 (12)	0.0015 (17)	0.0056 (16)	0.0016 (12)
O5	0.058 (3)	0.047 (2)	0.077 (3)	−0.005 (2)	0.005 (2)	−0.022 (2)
N1	0.033 (2)	0.034 (2)	0.0183 (17)	−0.0005 (17)	0.0000 (15)	0.0018 (16)
N2	0.033 (2)	0.038 (2)	0.033 (2)	0.0032 (17)	−0.0033 (18)	−0.0002 (17)
N3	0.038 (3)	0.061 (3)	0.113 (5)	−0.018 (3)	0.012 (3)	−0.036 (3)
N4	0.101 (5)	0.037 (3)	0.050 (3)	0.003 (3)	−0.014 (3)	−0.001 (2)
C1	0.035 (2)	0.036 (2)	0.0233 (17)	−0.001 (3)	0.001 (2)	0.0029 (18)
C2	0.036 (3)	0.034 (2)	0.0174 (19)	−0.002 (2)	0.0006 (18)	−0.0017 (19)
C3	0.031 (2)	0.032 (2)	0.026 (2)	−0.001 (2)	0.0001 (17)	−0.002 (2)
C4	0.031 (2)	0.027 (2)	0.022 (2)	0.0041 (18)	0.0027 (18)	−0.0020 (18)
C5	0.038 (3)	0.038 (3)	0.0168 (19)	−0.002 (2)	−0.0006 (19)	0.0007 (19)
C6	0.031 (2)	0.033 (2)	0.0201 (19)	0.0017 (19)	−0.0031 (17)	0.0007 (18)
C7	0.025 (2)	0.046 (3)	0.024 (2)	0.001 (2)	0.0020 (18)	−0.006 (2)
C8	0.047 (3)	0.032 (3)	0.056 (4)	−0.002 (2)	−0.003 (3)	−0.007 (3)
C9	0.036 (3)	0.044 (3)	0.070 (4)	0.004 (3)	0.000 (3)	−0.014 (3)
C10	0.036 (3)	0.046 (3)	0.056 (3)	−0.005 (2)	0.004 (2)	−0.015 (3)
C11	0.047 (3)	0.038 (3)	0.071 (4)	−0.001 (3)	−0.003 (3)	−0.016 (3)
C12	0.038 (3)	0.033 (3)	0.065 (4)	−0.004 (2)	−0.002 (3)	−0.016 (3)
C13	0.069 (4)	0.057 (4)	0.057 (4)	0.004 (3)	−0.005 (3)	−0.011 (3)
C14	0.37 (2)	0.047 (4)	0.079 (6)	−0.036 (8)	−0.019 (9)	−0.014 (4)
C15	0.096 (7)	0.053 (4)	0.079 (5)	0.029 (4)	0.008 (4)	0.011 (3)

### Geometric parameters (Å, º)

**Table:**

Cu1—O4^i^	1.955 (3)	C2—C4	1.381 (7)
Cu1—O2^ii^	1.966 (3)	C2—H2A	0.9300
Cu1—N2	1.999 (4)	C3—C6	1.384 (6)
Cu1—N1	2.052 (4)	C3—C4	1.388 (6)
Cu1—O5	2.396 (4)	C3—H3C	0.9300
O1—C7	1.226 (6)	C5—C6	1.384 (7)
O2—C7	1.277 (6)	C5—H5A	0.9300
O2—Cu1^iii^	1.966 (3)	C6—C7	1.511 (6)
O3—C1	1.228 (6)	C8—C9	1.362 (8)
O4—C1	1.281 (5)	C8—H8A	0.9300
O4—Cu1^iv^	1.955 (3)	C9—C10	1.390 (8)
O5—C13	1.225 (7)	C9—H9A	0.9300
N1—C5	1.332 (6)	C10—C11	1.387 (8)
N1—C2	1.349 (6)	C11—C12	1.368 (8)
N2—C8	1.330 (7)	C11—H11A	0.9300
N2—C12	1.346 (6)	C12—H12A	0.9300
N3—C10	1.337 (7)	C13—H13A	0.9300
N3—H3A	0.8600	C14—H14A	0.9600
N3—H3B	0.8600	C14—H14B	0.9600
N4—C13	1.322 (8)	C14—H14C	0.9600
N4—C14	1.428 (9)	C15—H15A	0.9600
N4—C15	1.440 (9)	C15—H15B	0.9600
C1—C4	1.506 (6)	C15—H15C	0.9600
			
O4^i^—Cu1—O2^ii^	178.46 (14)	N1—C5—H5A	118.4
O4^i^—Cu1—N2	88.29 (16)	C6—C5—H5A	118.4
O2^ii^—Cu1—N2	91.42 (16)	C3—C6—C5	118.5 (4)
O4^i^—Cu1—N1	90.10 (14)	C3—C6—C7	121.1 (4)
O2^ii^—Cu1—N1	90.16 (14)	C5—C6—C7	120.4 (4)
N2—Cu1—N1	177.93 (16)	O1—C7—O2	125.0 (5)
O4^i^—Cu1—O5	90.59 (16)	O1—C7—C6	120.1 (4)
O2^ii^—Cu1—O5	90.93 (16)	O2—C7—C6	114.9 (4)
N2—Cu1—O5	91.74 (16)	N2—C8—C9	124.2 (5)
N1—Cu1—O5	89.57 (16)	N2—C8—H8A	117.9
C7—O2—Cu1^iii^	114.6 (3)	C9—C8—H8A	117.9
C1—O4—Cu1^iv^	118.5 (3)	C8—C9—C10	119.9 (5)
C13—O5—Cu1	141.8 (5)	C8—C9—H9A	120.0
C5—N1—C2	117.7 (4)	C10—C9—H9A	120.0
C5—N1—Cu1	121.4 (3)	N3—C10—C11	120.7 (5)
C2—N1—Cu1	120.0 (3)	N3—C10—C9	123.3 (5)
C8—N2—C12	116.2 (5)	C11—C10—C9	116.0 (5)
C8—N2—Cu1	122.5 (3)	C12—C11—C10	120.5 (5)
C12—N2—Cu1	121.3 (4)	C12—C11—H11A	119.8
C10—N3—H3A	120.0	C10—C11—H11A	119.8
C10—N3—H3B	120.0	N2—C12—C11	123.0 (5)
H3A—N3—H3B	120.0	N2—C12—H12A	118.5
C13—N4—C14	120.0 (7)	C11—C12—H12A	118.5
C13—N4—C15	121.0 (6)	O5—C13—N4	125.5 (6)
C14—N4—C15	119.0 (7)	O5—C13—H13A	117.3
O3—C1—O4	126.0 (4)	N4—C13—H13A	117.3
O3—C1—C4	119.6 (4)	N4—C14—H14A	109.5
O4—C1—C4	114.4 (4)	N4—C14—H14B	109.5
N1—C2—C4	123.0 (4)	H14A—C14—H14B	109.5
N1—C2—H2A	118.5	N4—C14—H14C	109.5
C4—C2—H2A	118.5	H14A—C14—H14C	109.5
C6—C3—C4	119.1 (4)	H14B—C14—H14C	109.5
C6—C3—H3C	120.5	N4—C15—H15A	109.5
C4—C3—H3C	120.5	N4—C15—H15B	109.5
C2—C4—C3	118.4 (4)	H15A—C15—H15B	109.5
C2—C4—C1	120.1 (4)	N4—C15—H15C	109.5
C3—C4—C1	121.3 (4)	H15A—C15—H15C	109.5
N1—C5—C6	123.3 (4)	H15B—C15—H15C	109.5

Symmetry codes: (i) *x*−1/2, −*y*+1/2, −*z*+1; (ii) −*x*, *y*−1/2, −*z*+3/2; (iii) −*x*, *y*+1/2, −*z*+3/2; (iv) *x*+1/2, −*y*+1/2, −*z*+1.

### Hydrogen-bond geometry (Å, º)

**Table:**

*D*—H···*A*	*D*—H	H···*A*	*D*···*A*	*D*—H···*A*
N3—H3*A*···O3^v^	0.86	2.28	3.101 (7)	159
N3—H3*B*···O1^vi^	0.86	2.23	2.933 (7)	139

Symmetry codes: (v) *x*−1, *y*−1, *z*; (vi) −*x*−1, *y*−1/2, −*z*+3/2.
